# Phylogeographic Structure and Molecular Evolution of Squash Leaf Curl China Virus

**DOI:** 10.3390/v18070794

**Published:** 2026-07-19

**Authors:** Jingwen Yu, Xue Han, Yaqin Liu, Deliang Peng, Huan Peng, Houxiang Kang, Mingjun Li, Gentu Wu, Ling Qing, Wenkun Huang

**Affiliations:** 1 State Key Laboratory for Biology of Plant Diseases and Insect Pests, Institute of Plant Protection, Chinese Academy of Agricultural Sciences, Beijing 100193, China; jingwenyu1996@163.com (J.Y.); hanxue2729@163.com (X.H.);; 2 Chongqing Key Laboratory of Plant Disease Biology, College of Plant Protection, Southwest University, Chongqing 400715, China; 3 National Citrus Engineering Research Center, Southwest University, Chongqing 400712, China

**Keywords:** squash leaf curl China virus, population structure, genetic diversity, molecular variation

## Abstract

Squash leaf curl China virus (SLCCNV) is an important geminivirus that infects cucurbit crops and is widely distributed across Asia. To elucidate its population structure and molecular evolution, 101 DNA-A and 67 DNA-B strain sequences of SLCCNV that were publicly available from 2001 to 2024 were analyzed. The strains clustered into three major geographic clades, including South Asia, the Malay Archipelago, and Mainland Southeast Asia. Recombination analysis revealed breakpoints mainly concentrated in the *AC2* and *BC1* regions. Signals of positive selection were indicated for *AC4* and *AC5* by selection pressure analysis. Significant genetic differentiation among SLCCNV populations from different geographic origins, but frequent gene flow was observed between among populations from South Asia, the Malay Archipelago, and Mainland Southeast Asia. In addition, *AC5* and *AV2* exhibited high variability at both the nucleotide and amino acid levels, while *AC1*, *AC2*, and *AC3* were relatively conserved. Collectively, the evolutionary dynamics of SLCCNV are shaped by geographic isolation, recombination events, and differential selection pressures. This study provides important insights into the molecular evolution of SLCCNV and offers valuable guidance for region-specific surveillance, quarantine strategies, and the deployment of durable resistance against emerging viral variants.

## 1. Introduction

The family *Geminiviridae* comprises phytopathogenic viruses that cause significant yield losses in crops and are primarily transmitted by the whitefly *Bemisia tabaci* during its piercing-sucking feeding activity [1]. To date, the family *Geminiviridae* comprises 15 genera, among which begomoviruses represent the largest genus, encompassing over 445 recognized species [2,3]. Most begomovirus species possess a bipartite genome consisting of two single-stranded circular DNA molecules (~2.7 kb each), whereas a minority are monopartite [4].

Squash leaf curl China virus (SLCCNV), a bipartite begomovirus within the family *Geminiviridae*, harbors two circular single-stranded DNAs, DNA-A and DNA-B [5]. The DNA-A component encodes seven proteins (AV1, AV2, AC1, AC2, AC3, AC4, and AC5), while DNA-B encodes two proteins, BV1 and BC1 [6,7]. These viral proteins execute distinct functions during infection. For example, AC1 encodes the replication-associated protein (Rep), which initiates rolling-circle replication via site-specific cleavage [8]. AC2 functions as a transcriptional activator protein (TrAP), contributing to transcriptional activation, pathogenicity, and suppression of RNA silencing [9]. AC3 serves as a replication enhancer (REn), interacting with AC1 to promote efficient replication, whereas AC4 is involved in symptom development and viral movement [10]. AV1 encodes the coat protein responsible for genome encapsidation and whitefly-mediated transmission, while AV2 participates in viral movement [11]. On the DNA-B component, BV1 enhances virulence during infection, and BC1 acts as a movement protein mediating intracellular trafficking and systemic movement [12]. Additionally, AC5 has been reported to act as an RNA silencing suppressor and virulence determinant [7].

SLCCNV infects a broad range of cucurbit crops across South and Southeast Asia and can cause severe yield losses in squash, wax gourd, pumpkin and ash gourd [13,14,15,16,17]. A particularly severe outbreak on ash gourd (*Benincasa hispida*) in India resulted in 100% crop loss from 2012 to 2013 [17,18]. In China, the virus has been found to infect tomato, eggplant, and common bean, highlighting its progressively broadening host range [19,20,21].

The population structure, genetic diversity, and molecular evolution of plant viruses are closely associated with their epidemiology, geographical origin, host adaptation, and transmission vectors [22]. The population structure of several begomoviruses has been characterized. For example, recombination, positive selection, and environmental adaptation are key evolutionary forces shaping the tomato yellow leaf curl China virus (TYLCCNV) [23]. Characterization of maize yellow mosaic virus (MaYMV) has revealed geographic differentiation in its population structure and positive selection acting on the movement protein [24]. In recent years, research on SLCCNV has been confined to molecular characterization and geographic distribution [25,26]. Comprehensive studies on the population genetics and spatiotemporal dynamics of SLCCNV remain limited.

To elucidate the molecular evolution and ecological adaptation of SLCCNV strains, this study pursued the following objectives: (i) assess the geographic distribution and population genetic structure of SLCCNV; (ii) reconstruct phylogenetic relationships and detect coevolutionary signals among viral populations; (iii) identify patterns of genetic differentiation within SLCCNV populations. These findings will provide insights into the molecular evolution and ecological adaptation of SLCCNV.

## 2. Materials and Methods

### 2.1. Collection and Alignment of SLCCNV Strain Sequences

A total of 101 DNA-A strains and 67 DNA-B strains were retrieved from the NCBI database, covering the period from 2001 to 2024 (Supporting Information: Tables S1 and S2). The DNA-A strains originated from South Asia (18 from India; 1 from Bhutan; 1 from Bangladesh; 1 from Pakistan), the Malay Archipelago (6 from the Philippines; 5 from Indonesia; 7 from Malaysia), and Mainland Southeast Asia (40 from China; 12 from Thailand; 4 from Vietnam; 2 from Cambodia). The DNA-B strains were obtained from South Asia (15 from India; 1 from Bhutan; 1 from Bangladesh; 2 from Pakistan), the Malay Archipelago (1 from Indonesia; 6 from Malaysia), and Mainland Southeast Asia (35 from China; 1 from Thailand; 2 from Vietnam; 1 from Cambodia). The analysis encompassed full-length DNA-A genomes and seven open reading frames (ORFs) (*AV1*, *AV2*, *AC1*, *AC2*, *AC3*, *AC4*, *AC5*), as well as full-length DNA-B genomes and the associated *BC1* and *BV1* ORFs. Multiple sequence alignments were performed using the CLUSTALW algorithm in MEGA12 [27].

### 2.2. Recombination Signal Detection

SplitsTree4 v.4.13.1 [28] was used to detect recombination signals, with networks constructed using 1000 bootstrap replicates. RDP4 v.4.16 [29] was used to detect recombination events using seven algorithms (RDP, GENECONV, MaxChi, Chimaera, BOOTSCAN, SISCAN, 3Seq). Events supported by ≥4 methods and *p* < 1 × 10^−6^ were considered significant [30,31].

### 2.3. Phylogenetic Analysis of SLCCNV Strains

Multiple sequence alignments were generated with the ClustalW algorithm in MEGA12. Phylogenetic trees for DNA-A and DNA-B sequences were constructed using the maximum likelihood method in MEGA12, based on aligned nucleotide datasets. Evolutionary distances were computed using the Maximum Composite Likelihood model. Branch support was evaluated with 1000 bootstrap replicates. Pairwise nucleotide identities within and between phylogenetic groups were calculated using BioEdit 7.1.9 and SDT v1.2 software [32].

### 2.4. Selection Pressure and Neutrality Test Analysis

To assess the evolutionary dynamics of SLCCNV populations, Tajima’s D test [33] was applied to evaluate neutrality. Genetic differentiation among DNA-A and DNA-B populations was assessed using three statistical measures (Ks*, Z*, and Snn) [28], with *p*-values < 0.05 indicating significant differentiation. Gene flow among populations was estimated by calculating standardized variance of allele frequencies (Fst) and the number of migrants per generation (Nm) [24]. Populations with |Fst| > 0.33 or |Nm| < 1 were considered to have restricted gene flow, whereas |Fst| < 0.33 or |Nm| > 1 indicated frequent genetic exchange. The distribution of selection pressures across coding regions was further assessed by calculating the ratio of nonsynonymous to synonymous substitution rates (ω = dN/dS) using DnaSP 6 software [34].

### 2.5. Population Genetic Parameter Calculation

Based on phylogenetic groups and geographic origin, population genetic parameters were estimated for each coding region of DNA-A and DNA-B sequences using DnaSP 6. Insertions and deletions (InDels) were manually identified and tabulated based on sequence alignments. Nucleotide diversity (π) was estimated using a sliding window approach (window size = 100 nt, step size = 25 nt), and haplotype diversity (h) as well as average number of nucleotide differences were calculated to assess the distribution of genetic variation within populations [35].

## 3. Results

### 3.1. Recombination Patterns Were Observed in the SLCCNV Strains

**Figure 1:**
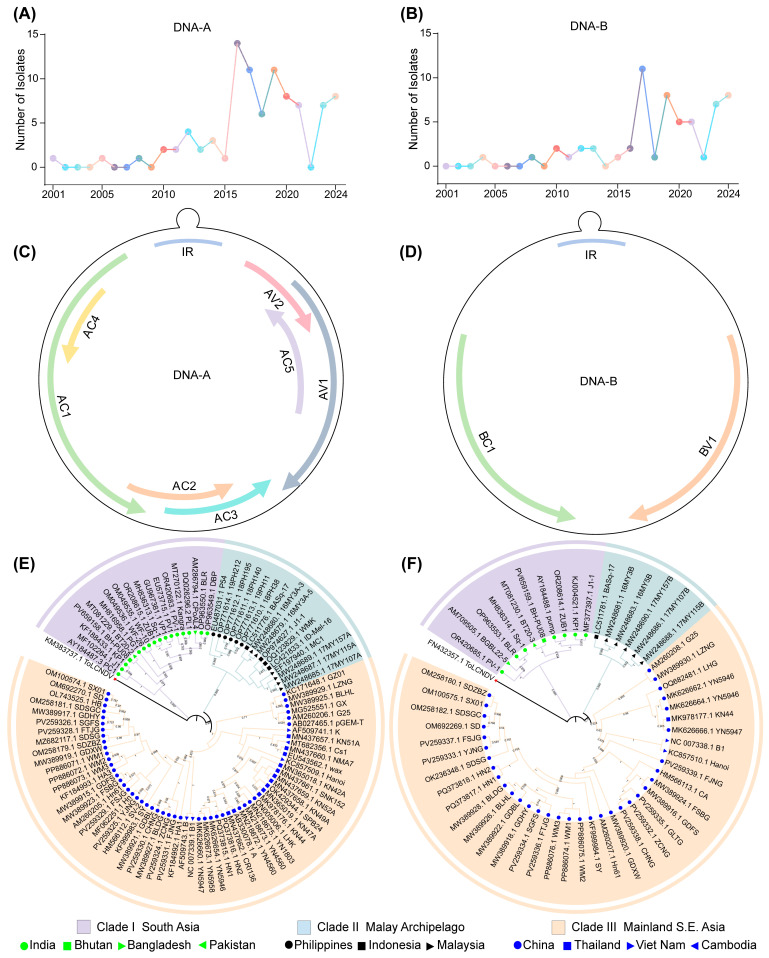
Temporal dynamics, genome organization, and phylogenetic analysis of SLCCNV. (**A**) Dynamics of SLCCNV DNA-A strains in 2001–2024. (**B**) Dynamics of SLCCNV DNA-B strains in 2001–2024. (**C**) Genome organization features of SLCCNV DNA-A. (**D**) Genome organization features of SLCCNV DNA-B. (**E**) Phylogenetic trees for DNA-A sequences were constructed using the maximum likelihood method in MEGA12. (**F**) Phylogenetic trees for DNA-B sequences were constructed using the maximum likelihood method in MEGA12. Different shapes and colors of the symbols represent different countries. Tomato leaf curl New Delhi virus (ToLCNDV DNA-A, NCBI accession number KM38373.1 and DNA-B, NCBI accession number FN432357.1) served as an outgroup.

A total of 101 DNA-A and 67 DNA-B sequences of SLCCNV strains collected between 2001 and 2024 were retrieved from GenBank, with strain numbers increasing notably after 2015 (Figure 1A–D; Supporting Information: Tables S1 and S2). Recombination analysis using SplitsTree 4 v.4.14.6 revealed extensive reticulation between DNA-A and DNA-B components, indicative of frequent recombination events. The constructed network further resolved three major clades corresponding to geographic origins: South Asia, the Malay Archipelago, and Mainland Southeast Asia (Supporting Information: Figure S1A,B).

To characterize recombination events in the SLCCNV strains, RDP4 analysis identified seven recombinant events among DNA-A strains, each supported by at least four detection methods (*p* < 0.05), and 14 recombinants among DNA-B strains. Most Recombination breakpoints (85.7%) clustered within the *AC2* region of DNA-A (nt 1190–1594) and the *BC1* region of DNA-B (nt 1337–2182), representing 92.9% of total breakpoints (Table 1, Supporting Information: Figure S2).

### 3.2. SLCCNV Strains Cluster into Three Genetic Groups

To clarify phylogenetic relationships of SLCCNV strains, full-genome sequences of 94 DNA-A and 53 DNA-B strains (excluding recombinant sequences) were analyzed using MEGA12. All SLCCNV DNA-A and DNA-B strains clustered into three phylogenetic groups broadly corresponding to geographic origin: Clade I (South Asia: India, Bhutan, Bangladesh, Pakistan), Clade II (Malay Archipelago: Philippines, Indonesia, Malaysia), and Clade III (Mainland Southeast Asia: China, Thailand, Vietnam, Cambodia) (Figure 1E,F). Furthermore, phylogenetic grouping was independent of host species (Supporting Information: Figure S3).

### 3.3. Sequence Identity Analysis in SLCCNV Strains

**Figure 2:**
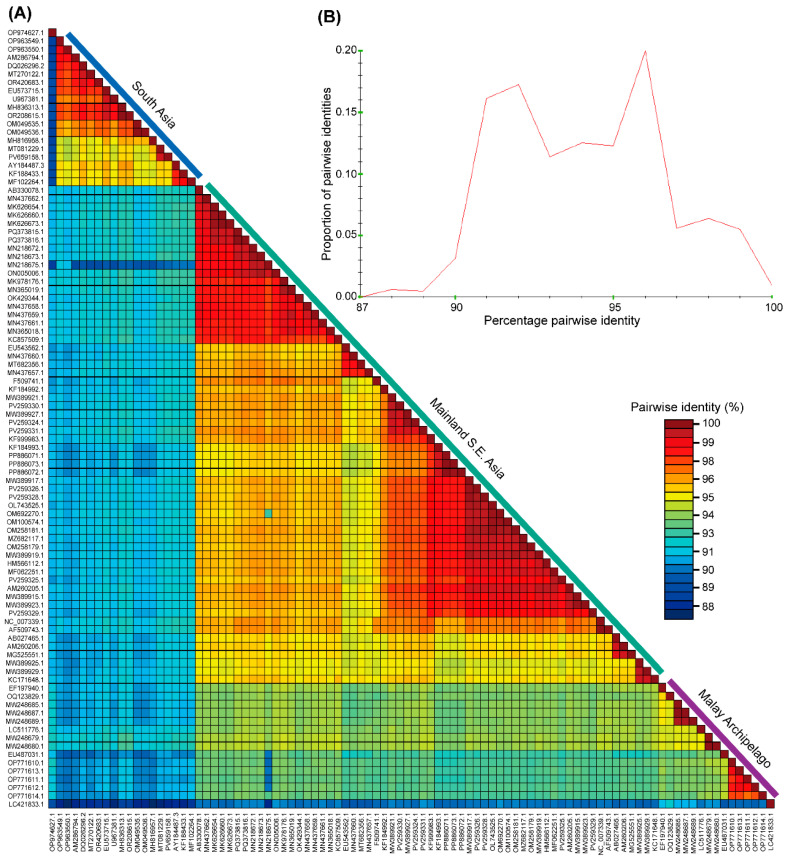
Nucleotide sequence identity analysis of SLCCNV DNA-A strains. (**A**) Pairwise nucleotide sequence identity among SLCCNV DNA-A strains. The color scale indicates the percentage of nucleotide identity between sequences. (**B**) Pairwise sequence identity distribution of SLCCNV strains.

**Figure 3:**
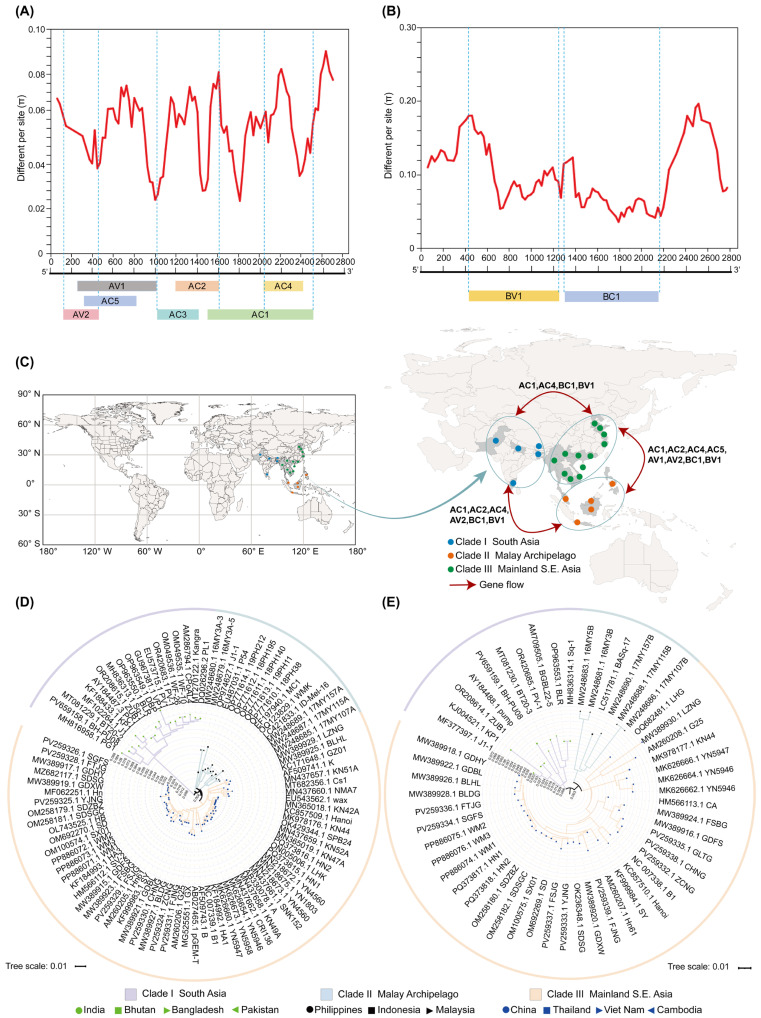
Genetic characteristics of SLCCNV. (**A**) Distribution of nucleotide diversity (π) across different genetic regions in 94 SLCCNV DNA-A sequences. (**B**) Distribution of nucleotide diversity (π) across different genetic regions in 53 SLCCNV DNA-B sequences. The nucleotide diversity (Y-axis) was plotted against nucleotide position (X-axis) using DnaSP6 with a 100-nucleotide (nt) sliding window and a 25-nt step size. (**C**) Global phylogeographic distribution and gene flow of SLCCNV strains. Colors correspond to the three major clades. (**D**) Phylogenetic trees for AV1 sequences were constructed using the maximum likelihood method in MEGA12. (**E**) Phylogenetic trees for BV1 sequences were constructed using the maximum likelihood method in MEGA12. Different shapes and colors of the symbols represent different countries.

Nucleotide sequence identity was evaluated across phylogenetic groups of SLCCNV strains, with nucleotide identities of 87–100% for DNA-A strains and 81–100% for DNA-B strains (Figure 2A,B; Supporting Information: Figure S4A,B). The highest variation in *AC5* and *AV2* was found within DNA-A coding regions, with the lowest in *AC2* by nucleotide diversity (π) analysis (Figure 3A). Similarly, DNA-B coding regions *BC1* and *BV1* also exhibited high nucleotide variability (Figure 3B).

The degree of variation in different coding regions differed between the nucleotide and amino acid levels. Within DNA-A strains, AC5 (72.25%) and AV2 (74.86%) had the lowest amino acid identity, significantly lower than AC1 (90.74%), AC2 (91.94%), AC3 (94.39%), AC4 (92.40%), and AV1 (98.45%) (Table 2). A similar pattern was observed at the nucleotide level, with *AC5* (77.11%) and *AV2* (76.06%) being the most variable regions, whereas *AC4* (96.90%) was the most conserved. In DNA-B strains, BC1 had slightly lower amino acid (87.86%) and nucleotide identity (87.84%) compared with BV1. Analysis of mutation types revealed that nonsynonymous substitutions predominated in AC2, AC5, AV1, and AV2. Insertions/deletions (InDels) were detected in AC3, AC5, AV1, and AV2, and twice at the terminal regions of AC1 (Supporting Information: Table S3). In DNA-B, amino acid variation in BV1 was primarily nonsynonymous, and two InDels were detected within the BC1.

### 3.4. Analysis of Selection Pressure and Neutrality on SLCCNV Encoded Proteins

Nucleotide and haplotype diversity were assessed across SLCCNV strains. *AC3* exhibited the lowest haplotype diversity (0.977 ± 0.009), whereas *AC1* and *AV2* had the highest (0.996 ± 0.002) (Table 3). Nucleotide diversity for *AC1* (0.08997 ± 0.30602), *AC2* (0.08286 ± 0.29108), and *AC3* (0.06065 ± 0.12190) were all below 0.1. Tajima’s D was negative in Clades I (South Asia) and Clades II (Malay Archipelago), indicating that DNA-A strains follow a neutral evolutionary model. The mean dN/dS ratio showed that seven genes (*AC1*, *AC2*, *AC3*, *AV1*, *AV2*, *BV1*, *BC1*) were under negative or purifying selection, whereas *AC4* and *AC5* were under positive selection (dN/dS > 1).

### 3.5. Heterogeneous Genetic Differentiation of SLCCNV Across Geographic Regions

Population genetic analyses using Ks*, Z*, and Snn statistics revealed significant differentiation among SLCCNV populations across geographic regions (*p* < 0.05) (Table 4). Gene flow estimates (Fst and Nm) indicated frequent exchange among all three geographic clades (South Asia, Malay Archipelago, and Mainland Southeast Asia) across different coding regions (|Fst| < 0.33, |Nm| > 1) (Figure 3C). Considering the roles of *AV1* and *BV1* in viral transmission, phylogenetic trees were constructed based on *AV1* sequences from DNA-A strains and *BV1* sequences from DNA-B strains. Malay Archipelago and Mainland Southeast Asia strains formed distinct clusters, supporting significant genetic differentiation between geographic populations (Figure 3D,E).

## 4. Discussion

The genus *Begomovirus* (family Geminiviridae) comprises a large and genetically diverse group of plant viruses responsible for substantial crop yield reductions in tropical and subtropical regions. SLCCNV has emerged as an important pathogen of cucurbitaceous crops across South and Southeast Asia. Recent reports further indicate an expansion of its host range to include solanaceous and leguminous species [19,26,36]. Despite its economic importance, the population genetic structure and evolutionary processes underlying SLCCNV diversity remain insufficiently characterized. In this study, a comprehensive population genomic analysis of SLCCNV strains revealed its phylogeographic structure, recombination patterns, and adaptive evolutionary dynamics.

Phylogenetic reconstruction resolved SLCCNV strains into three major clades corresponding to South Asia (Clade I), the Malay Archipelago (Clade II), and Mainland Southeast Asia (Clade III). This geographic structuring aligns with patterns observed in other begomoviruses, such as TYLCCNV and tobacco curly shoot virus (TbCSV), wherein spatial separation serves as a primary driver of genetic divergence [37,38]. Notably, the absence of host-associated phylogenetic clustering suggests that host shifts are unlikely to be a dominant factor shaping SLCCNV diversification. These findings imply that viral dispersal is more strongly constrained by geographic distance and vector ecology than by host availability [39,40,41].

Recombination and natural selection represent major drivers of genetic variation within the genus *Begomovirus* and are frequently associated with shifts in virulence and host range [42,43]. In SLCCNV, multiple recombination events were detected across both genomic components, with breakpoints concentrated in the *AC2* and *BC1* regions. These loci encode the transcriptional activator protein and the movement protein, suggesting that both mechanistic susceptibility to template switching and selective retention of advantageous variants may contribute to hotspot formation. Consistent with this observation, most coding regions were subject to strong purifying selection, reflecting functional constraints on core viral processes. By contrast, *AC4* and *AC5* exhibited signatures of positive selection, indicative of adaptive divergence potentially driven by interactions with host defense pathways. These findings underscore the interplay between recombination and selection in shaping SLCCNV evolution, while also indicating that recombination frequency may be underestimated due to uneven sampling.

Analysis of sequence conservation across viral proteins revealed distinct evolutionary constraints among genomic regions. Within DNA-A, *AC5* and *AV2* displayed the lowest levels of amino acid and nucleotide conservation, consistent with their roles as accessory proteins that tolerate greater sequence flexibility [44]. Conversely, *AC1*, *AC2*, and *AC3* exhibited markedly higher conservation, reflecting the stringent structural and functional constraints imposed by their roles in replication and transcriptional regulation [45]. In DNA-B, the movement protein BC1 demonstrated slightly reduced conservation relative to BV1, potentially reflecting host-specific adaptations in systemic trafficking functions [46,47].

Population genetic analyses revealed significant genetic differentiation among SLCCNV populations from South Asia, the Malay Archipelago, and Mainland Southeast Asia, while estimates of gene flow indicated ongoing genetic exchange among these regional clades. Given the substantial geographic barriers separating these regions, such connectivity is unlikely to be explained solely by the natural dispersal capacity of the whitefly vector. Instead, long-distance movement of infected planting materials through agricultural trade, passive transport of viruliferous whiteflies, and recurrent mixed infections that promote viral recombination likely contribute to the observed genetic connectivity [48,49]. Neutrality tests further indicated contrasting demographic histories, with predominantly negative Tajima’s D values in Clades I and II consistent with population expansion or recent selective sweeps, whereas Clade III showed more heterogeneous patterns, including positive values in some regions that may reflect balancing selection or more complex dynamics. Consistent with previous population genetic studies of other begomoviruses, including TYLCCNV and MaYMV, these findings suggest that the population structure of SLCCNV may be shaped by the combined effects of geographic isolation, recombination, vector ecology, and human-mediated dispersal [23,24,50].

Several methodological limitations should be acknowledged. First, the available SLCCNV sequence data exhibit geographic and temporal heterogeneity, with most strains originating from China while regions such as South Asia and the Malay Archipelago remain relatively undersampled. Furthermore, although bioinformatic analyses identified positively selected sites and recombination hotspots, experimental validation of the functional consequences of these genetic variations remains an important avenue for future investigation. Future studies would benefit from expanded field sampling, particularly in under-represented geographic regions, together with time-aware evolutionary analyses and functional assays to clarify the biological significance of genetic variation and improve epidemiological inference.

## 5. Conclusions

In summary, this study establishes a population genomic framework for understanding SLCCNV evolutionary dynamics across Asia between 2001 and 2024. Results demonstrate that SLCCNV populations are geographically structured, shaped by recombination and heterogeneous selection pressures, with distinct evolutionary patterns across genomic components. Specifically, three phylogeographic clades are delineated. Recombination events concentrate in *AC2* and *BC1* regions, whereas positive selection acts on *AC4* and *AC5*. Significant genetic differentiation exists among geographic populations; however, gene flow between South Asia and the Malay Archipelago, as well as Mainland Southeast Asia, indicates regional connectivity. Geographic isolation, recombination, and differential selection collectively shape SLCCNV evolutionary trajectories. These insights contribute to understanding *begomovirus* evolution and inform region-specific surveillance and phytosanitary measures to reduce cross-regional virus dissemination.

## Figures and Tables

**Table 1:** Putative recombination events in SLCCNV strains.

			Putative Parent			Detection Method ^b^						
	Event	Putative Recombinant	Major	Minor	Breakpoint ^a^	R	G	B	M	C	S	T
DNA-A	1	J1 MN594504.1	WMK OQ123829.1	Unknown (SV-1) LC417095.1	56–1543	++	++	++	++	++	++	++
	2	SV-1 LC417095.1	WMK OQ123829.1	Unknown (PV1) EU573715.1	56–1544	++	++	++	++	++	++	++
	3	Vir-6569 MK064240.1	Cs1 MT682356.1	Unknown (SX01) OM100574.1	998–1478	++	++	++	++	++	++	++
	4	TMK OP963548.1	PV1 EU573715.1	Unknown (WMK) OQ123829.1	1473–1864	++	++	++	++	+	+	++
	5	Pum JN587811.1	Kangra MT270122.1	Unknown (16MY5A) MW248682.1	1541–2543	+	-	-	++	++	+	++
	6	16MY5A MW248682.1	PV-1 OR420683.1	Unknown (PB1) OR135585.1	932–2564	++	++	++	++	++	++	++
	7	WF-32 OM049537.1	PV1 EU573715.1	PG1 MH816957.1	1502–1980	++	+	+	+	+	+	++
DNA-B	1	PG1 MH816957.1	DTMK OP963551.1	Unknown (Varanasi) GU967382.1	1329–1925	-	++	-	++	-	++	++
	2	DTMK OP963551.1	BLR OP963553.1	GZ01 KC171649.1	710–1267	++	++	++	++	++	++	++
	3	Pum JN624306.1	BGBL22-5 AM709505.1	ZUB1 OR208614.1	1333–2172	++	++	++	++	++	++	++
	4	KM2 OR860426.1	KP1 KJ004521.1	Unknown (CPoBL2) AM778959.1	1–2498	++	++	++	++	++	++	++
	5	Varanasi GU967382.1	CPoBL2 AM778959.1	BLR OP963553.1	63–2182	++	++	++	++	-	++	++
	6	J1 MN594505.1	J1-1 MF377397.1	Unknown (BLR) OP963553.1	1968–2269	++	++	-	++	++	+	++
	7	17MY85B MW248684.1	17MY157B MW248690.1	Unknown (GX2017) MG525552.1	28–2580	++	++	++	+	++	-	++
	8	DBP OP963552.1	CPoBL2 AM778959.1	BLR OP963553.1	1535–2425	-	-	++	++	-	++	++
	9	GX2017 MG525552.1	LHG OQ682481.1	FJNG PV259339.1	215–1505	+	-	-	++	++	++	++
	10	GZ01 KC171649.1	YN5947 MK626666.1	GDFS MW389916.1	1210–1380	-	-	-	++	++	+	+
	11	Cs1 MT682357.1	Hanoi KC857510.1	17MY157B MW248690.1	346–1185	-	-	-	+	++	++	+
	12	CPoBL2 AM778959.1	BGBL22-5 AM709505.1	DTMK OP963551.1	2150–2643	++	+	++	+	+	++	+
	13	Varanasi-1 FJ859881.1	BGBL22-5 AM709505.1	SDSGC OM258182.1	2056–2122	++	+	+	-	-	-	+
	14	Hn MF062252.1	Hn61 AM260207.1	B1 NC_007338.1	998–1602	+	-	-	++	+	-	+

^a^ Recombination breakpoint. ^b^ Seven algorithms were used: RDP (R), GENECONV (G); BootScan (B), Maximum χ-Square (M), Chimaera (C), SisScan (S), and 3Seq (T). Statistical significance for recombination: + indicates 10^−6^ < *p* value ≤ 0.05, ++ indicates *p* value ≤ 10^−6^ and - indicates not significant (*p* value > 0.05).

**Table 2:** Sequence identities (ID), insertion or deletion events (InDels), and site nucleotide mutations in individual genes or proteins encoded by SLCCNV strains.

Genomic Region ^a^	Amino Acids (aa)			Nucleotide (nt)		Mutations ^b^	
	Length (aa)	ID (%)	InDels	Length (nt)	ID (%)	Syn	Non
AC1 (Rep)	238–373	90.74	I, II	717–1122	90.03	228	220
AC2 (TrAP)	133–135	91.94	None	402–408	95.50	43	121
AC3 (Ren)	104–137	94.39	III	351–414	95.24	70	91
AC4 (SD)	58	92.40	None	177	96.90	11	34
AC5 (VSR)	104–210	72.25	IV	315–633	77.11	21	170
AV1 (CP)	251–256	98.45	V	756–771	96.19	160	244
AV2 (Pre-CP)	111–161	74.86	VI	336–486	76.06	37	151
BC1 (MP)	269–305	87.86	VII, VIII	810–918	87.84	208	266
BV1 (NSP)	184–268	92.80	None	555–807	92.62	188	236

^a^ AC1 = Replication-associated protein, AC2 = Transcriptional activator protein, AC3 = Replication enhancer protein, AC4 = Disease symptom determinants, AC5 = RNA silencing suppressor and virulence determinant protein, AV1 = Coat protein, AV2 = Pre-coat protein, BC1 = Movement protein, BV1= Promotion of virulence during the infection. ^b^ Number of nucleotide mutations. Syn = synonymous and Non = nonsynonymous.

**Table 3:** Genetic parameters, neutrality test, and selection pressure on SLCCNV strains subpopulations based on geographic origin.

Genomic Region ^a^	Population	*N* ^b^	Haplotype Diversity	Nucleotide Diversity	Tajima’s D ^c^	dN/dS ^d^
AC1 (Rep)	All (*n* = 94)	80	0.996 ± 0.002	0.08997 ± 0.30602	−2.41751 (**)	0.25
	Clade I (*n* = 18)	13	0.954 ± 0.034	0.12360 ± 0.25891	−2.23190 (**)	0.48
	Clade II (*n* = 16)	15	0.992 ± 0.025	0.06907 ± 0.09378	−1.15208 (ns)	0.18
	Clade III (*n* = 60)	53	0.995 ± 0.004	0.06371 ± 0.20009	−2.43701 (**)	0.25
AC2 (TrAP)	All (*n* = 94)	72	0.993 ± 0.003	0.08286 ± 0.29108	−2.43936 (**)	0.74
	Clade I (*n* = 18)	13	0.989 ± 0.031	0.02618 ± 0.03882	−1.42770 (ns)	0.67
	Clade II (*n* = 16)	17	0.982 ± 0.026	0.12220 ± 0.24839	−2.14110 (**)	0.94
	Clade III (*n* = 60)	44	0.988 ± 0.006	0.06457 ± 0.21444	−2.48188 (**)	0.76
AC3 (Ren)	All (*n* = 94)	67	0.977 ± 0.009	0.06065 ± 0.12190	−1.69596 (ns)	0.32
	Clade I (*n* = 18)	16	0.987 ± 0.023	0.03135 ± 0.05076	−1.58043 (ns)	0.53
	Clade II (*n* = 16)	13	0.981 ± 0.031	0.09051 ± 0.14891	−1.73446 (ns)	0.35
	Clade III (*n* = 60)	41	0.970 ± 0.013	0.02921 ± 0.04905	−1.40344 (ns)	0.27
AC4 (SD)	All (*n* = 94)	55	0.981 ± 0.005	0.09963 ± 0.36444	−2.46673 (**)	1.12
	Clade I (*n* = 18)	12	0.935 ± 0.041	0.12304 ± 0.25624	−2.19926 (**)	1.47
	Clade II (*n* = 16)	14	0.983 ± 0.028	0.04543 ± 0.06981	−1.46725 (ns)	1.17
	Clade III (*n* = 60)	29	0.959 ± 0.011	0.09950 ± 0.37991	−2.62156 (ns)	0.99
AC5 (VSR)	All (*n* = 94)	64	0.985 ± 0.006	0.26409 ± 0.31843	−0.58291 (ns)	1.12
	Clade I (*n* = 18)	16	0.987 ± 0.023	0.18486 ± 0.41808	−2.38188 (**)	1.06
	Clade II (*n* = 16)	14	0.983 ± 0.028	0.04078 ± 0.05362	−1.03000 (ns)	5.10
	Clade III (*n* = 60)	34	0.965 ± 0.013	0.03864 ± 0.05261	−0.91627 (ns)	3.61
AV1 (CP)	All (*n* = 94)	74	0.994 ± 0.003	0.15241 ± 0.35818	−1.96550 (*)	0.41
	Clade I (*n* = 18)	16	0.987 ± 0.023	0.09778 ± 0.23110	−2.45806 (**)	0.64
	Clade II (*n* = 16)	14	0.983 ± 0.028	0.03911 ± 0.05160	−1.04920 (ns)	0.06
	Clade III (*n* = 60)	45	0.988 ± 0.006	0.15836 ± 0.29448	−1.65464 (ns)	0.69
AV2 (Pre-CP)	All (*n* = 94)	80	0.996 ± 0.002	0.52620 ± 0.53823	−0.07658 (ns)	0.91
	Clade I (*n* = 18)	14	0.967 ± 0.030	0.18097 ± 0.40091	−2.33945 (**)	0.90
	Clade II (*n* = 16)	15	0.992 ± 0.025	0.28255 ± 0.52717	−2.03394 (*)	0.84
	Clade III (*n* = 60)	51	0.995 ± 0.004	0.53725 ± 0.51693	0.14091 (ns)	0.96
BC1 (MP)	All (*n* = 53)	44	0.993 ± 0.005	0.19036 ± 0.41487	−1.96736 (*)	0.50
	Clade I (*n* = 10)	8	0.956 ± 0.059	0.05425 ± 0.06317	−0.70244 (ns)	0.07
	Clade II (*n* = 6)	5	0.933 ± 0.122	0.06769 ± 0.06833	−0.06090 (ns)	0.07
	Clade III (*n* = 37)	31	0.989 ± 0.009	0.22655 ± 0.43355	−1.81424 (*)	0.72
BV1 (NSP)	All (*n* = 53)	46	0.994 ± 0.005	0.18359 ± 0.44231	−2.12478 (*)	0.64
	Clade I (*n* = 10)	8	0.956 ± 0.059	0.18963 ± 0.29616	−1.80196 (*)	0.74
	Clade II (*n* = 6)	6	1.000 ± 0.096	0.31178 ± 0.39245	−1.33843 (ns)	0.54
	Clade III (*n* = 37)	32	0.991 ± 0.009	0.13025 ± 0.36601	−2.44405 (**)	0.25

^a^ AC1 = Replication-associated protein, AC2 = Transcriptional activator protein, AC3 = Replication enhancer protein, AC4 = Disease symptom determinants, AC5 = RNA silencing suppressor and virulence determinant protein, AV1 = Coat protein, AV2 = Pre-coat protein, BC1 = Movement protein, BV1= Promotion of virulence during the infection. ^b^ Number of haplotypes. ^c^ ns = not significant (*p* > 0.05), * indicate *p* < 0.05, ** indicate *p* < 0.01. ^d^ Nonsynonymous/synonymous ratio (dN/dS) < 1 (negative selection), dN/dS = 1 (neutral evolution), and dN/dS > 1 (positive selection).

**Table 4:** Measurement of genetic differentiation SLCCNV strains based on geographic origin.

Genomic Region ^a^	Comparisons	Ks * (*p* Value ^b^)	Z * (*p* Value ^b^)	Snn (*p* Value ^b^)	Fst ^c^	Nm ^d^
AC1 (Rep)	Clade I (*n* = 18) versus Clade II (*n* = 16)	3.50888 (0.0000 ***)	4.85026 (0.0000 ***)	0.97059 (0.0000 ***)	0.19862	2.02
	Clade I (*n* = 18) versus Clade III (*n* = 60)	3.29400 (0.0000 ***)	6.62294 (0.0000 ***)	0.97436 (0.0000 ***)	0.22424	1.73
	Clade II (*n* = 16) versus Clade III (*n* = 60)	3.44938 (0.0000 ***)	6.67794 (0.0000 ***)	0.98684 (0.0000 ***)	0.23808	1.60
AC2 (TrAP)	Clade I (*n* = 18) versus Clade II (*n* = 16)	2.94546 (0.0000 ***)	4.92823 (0.0000 ***)	0.79293 (0.0030 **)	0.19222	2.10
	Clade I (*n* = 18) versus Clade III (*n* = 60)	2.39109 (0.0000 ***)	6.50911 (0.0000 ***)	1.00000 (0.0000 ***)	0.45071	0.61
	Clade II (*n* = 16) versus Clade III (*n* = 60)	2.60453 (0.0000 ***)	6.81203 (0.0000 ***)	0.98734 (0.0000 ***)	0.14292	3.00
AC3 (Ren)	Clade I (*n* = 18) versus Clade II (*n* = 16)	2.56062 (0.0000 ***)	4.73879 (0.0000 ***)	1.00000 (0.0000 ***)	0.36499	0.87
	Clade I (*n* = 18) versus Clade III (*n* = 60)	2.13830 (0.0000 ***)	6.60721 (0.0000 ***)	1.00000 (0.0000 ***)	0.63227	0.29
	Clade II (*n* = 16) versus Clade III (*n* = 60)	2.54404 (0.0000 ***)	6.65855 (0.0000 ***)	1.00000 (0.0000 ***)	0.36704	0.86
AC4 (SD)	Clade I (*n* = 18) versus Clade II (*n* = 16)	2.15290 (0.0000 ***)	4.95924 (0.0000 ***)	1.00000 (0.0000 ***)	0.14240	3.01
	Clade I (*n* = 18) versus Clade III (*n* = 60)	1.99564 (0.0000 ***)	6.74982 (0.0000 ***)	0.94338 (0.0000 ***)	0.10171	4.42
	Clade II (*n* = 16) versus Clade III (*n* = 60)	2.15290 (0.0000 ***)	4.95924 (0.0000 ***)	1.00000 (0.0000 ***)	0.14240	3.01
AC5 (VSR)	Clade I (*n* = 18) versus Clade II (*n* = 16)	2.83643 (0.0000 ***)	4.69172 (0.0000 ***)	0.97059 (0.0000 ***)	0.84108	0.09
	Clade I (*n* = 18) versus Clade III (*n* = 60)	2.46647 (0.0000 ***)	6.54332 (0.0000 ***)	0.97835 (0.0000 ***)	0.84526	0.09
	Clade II (*n* = 16) versus Clade III (*n* = 60)	2.40992 (0.0000 ***)	6.68095 (0.0000 ***)	1.00000 (0.0000 ***)	0.30352	1.15
AV1 (CP)	Clade I (*n* = 18) versus Clade II (*n* = 16)	2.63987 (0.0000 ***)	4.68293 (0.0000 ***)	0.97059 (0.0000 ***)	0.50094	0.50
	Clade I (*n* = 18) versus Clade III (*n* = 60)	2.90547 (0.0000 ***)	6.61566 (0.0000 ***)	0.98718 (0.0000 ***)	0.36052	0.89
	Clade II (*n* = 16) versus Clade III (*n* = 60)	3.30675 (0.0000 ***)	6.71370 (0.0000 ***)	1.00000 (0.0000 ***)	0.13991	3.07
AV2 (Pre-CP)	Clade I (*n* = 18) versus Clade II (*n* = 16)	2.94193 (0.0000 ***)	4.85203 (0.0000 ***)	0.97059 (0.0000 ***)	0.19735	2.03
	Clade I (*n* = 18) versus Clade III (*n* = 60)	4.00309 (0.0000 ***)	6.64058 (0.0000 ***)	0.98701 (0.0000 ***)	0.41618	0.70
	Clade II (*n* = 16) versus Clade III (*n* = 60)	4.35847 (0.0000 ***)	6.74824 (0.0000 ***)	0.93333 (0.0000 ***)	0.30047	1.16
BC1 (MP)	Clade I (*n* = 10) versus Clade II (*n* = 6)	3.72115 (0.0000 ***)	3.22690 (0.0000 ***)	1.00000 (0.0010 **)	0.32739	1.03
	Clade I (*n* = 10) versus Clade III (*n* = 37)	4.05877 (0.0000 ***)	5.76297 (0.0000 ***)	1.00000 (0.0000 ***)	0.17802	2.31
	Clade II (*n* = 6) versus Clade III (*n* = 37)	4.11281 (0.0000 ***)	5.65839 (0.0000 ***)	0.98837 (0.0000 ***)	0.17385	2.38
BV1 (NSP)	Clade I (*n* = 10) versus Clade II (*n* = 6)	4.10627 (0.0000 ***)	3.35998 (0.0000 ***)	0.93750 (0.0020 **)	0.09756	4.63
	Clade I (*n* = 10) versus Clade III (*n* = 37)	3.56271 (0.0000 ***)	5.69332 (0.0000 ***)	0.95745 (0.0000 ***)	0.25208	1.48
	Clade II (*n* = 6) versus Clade III (*n* = 37)	3.59772 (0.0000 ***)	5.63883 (0.0000 ***)	0.97674 (0.0000 ***)	0.14109	3.04

^a^ AC1 = Replication-associated protein, AC2 = Transcriptional activator protein, AC3 = Replication enhancer protein, AC4 = Disease symptom determinants, AC5 = RNA silencing suppressor and virulence determinant protein, AV1 = Coat protein, AV2 = Pre-coat protein, BC1 = Movement protein, BV1= Promotion of virulence during the infection. ^b^ Probability (*p* value) obtained by the permutation (PM) test with 1000 replicates, * indicate 0.01 < *p* < 0.05, ** indicate *p* < 0.01, and *** indicate *p* < 0.001. ^c^ Fst = standardized variance of allele frequencies across populations. ^d^ Nm = migration rate. |Fst| > 0.33 or |Nm| < 1 suggests infrequent gene flow; |Fst| < 0.33 or |Nm| > 1 suggests frequent gene flow.

## Data Availability

The data that support the findings of this study are available upon request from the corresponding author.

## Supplementary Materials

The following supporting information can be downloaded at: https://www.mdpi.com/article/10.3390/v18070794/s1, Table S1 Information for DNA-A strains of SLCCNV used in this study. Table S2 Information for DNA-B strains of SLCCNV used in this study. Table S3 InDels events in individual proteins of SLCCNV strains. Figure S1 Split network analysis of SLCCNV strains. (A) Split network analysis of 101 SLCCNV DNA-A strains. (B) Split network analysis of 67 SLCCNV DNA-B strains. Figure S2 Putative recombination breakpoints in the DNA-A (A) and DNA-B (B) components of SLCCNV strains. Figure S3 Phylogenetic trees of SLCCNV strains colored by host species. (A) Maximum likelihood phylogenetic tree was constructed using MEGA12 based on 94 SLCCNV DNA-A strains. (B) Maximum likelihood phylogenetic tree constructed using MEGA12 based on 53 SLCCNV DNA-B strains. Different colors are represented host. Figure S4 Nucleotide sequence identity analysis of SLCCNV DNA-B strains. (A) Pairwise nucleotide sequence identity among SLCCNV DNA-B strains. The color scale indicates the percentage of nucleotide identity between sequences. (B) Pairwise sequence identity distribution of SLCCNV DNA-B strains.
