# Supplementary material for: Phylogeographic Structure and Molecular Evolution of Squash Leaf Curl China Virus
Source: Viruses. 2026 Jul 19;18(7):794. doi: 10.3390/v18070794 (PMC13431508; doi:10.3390/v18070794)
Supplement: Supplementary file 1 [file viruses-18-00794-s001.zip › Supplementary Figures.pdf]

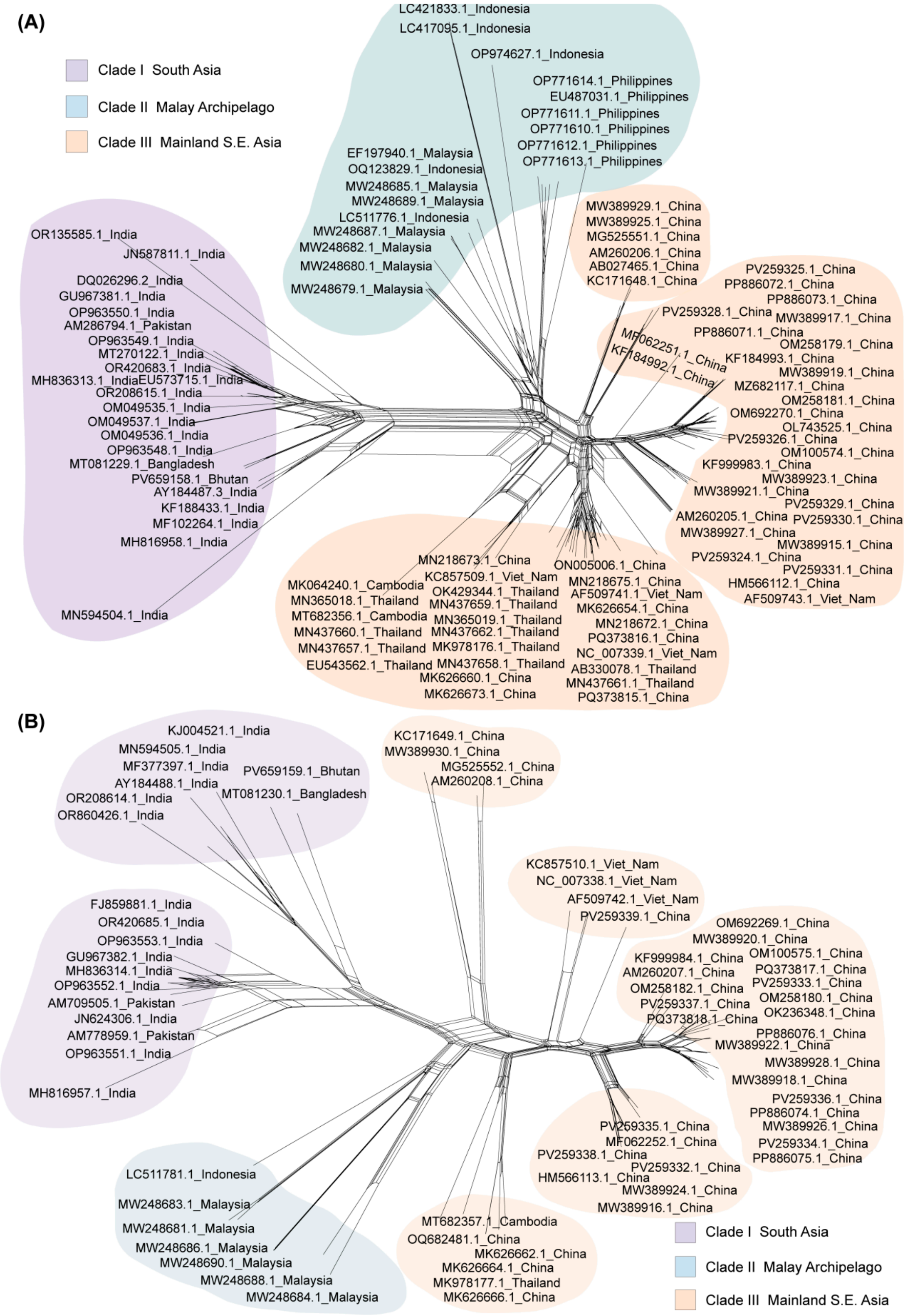

Figure S1. Split network analysis of SLCCNV strains. (A) Split network analysis of 101 SLCCNV DNA-A strains. (B) Split network analysis of 67 SLCCNV DNA-B strains.

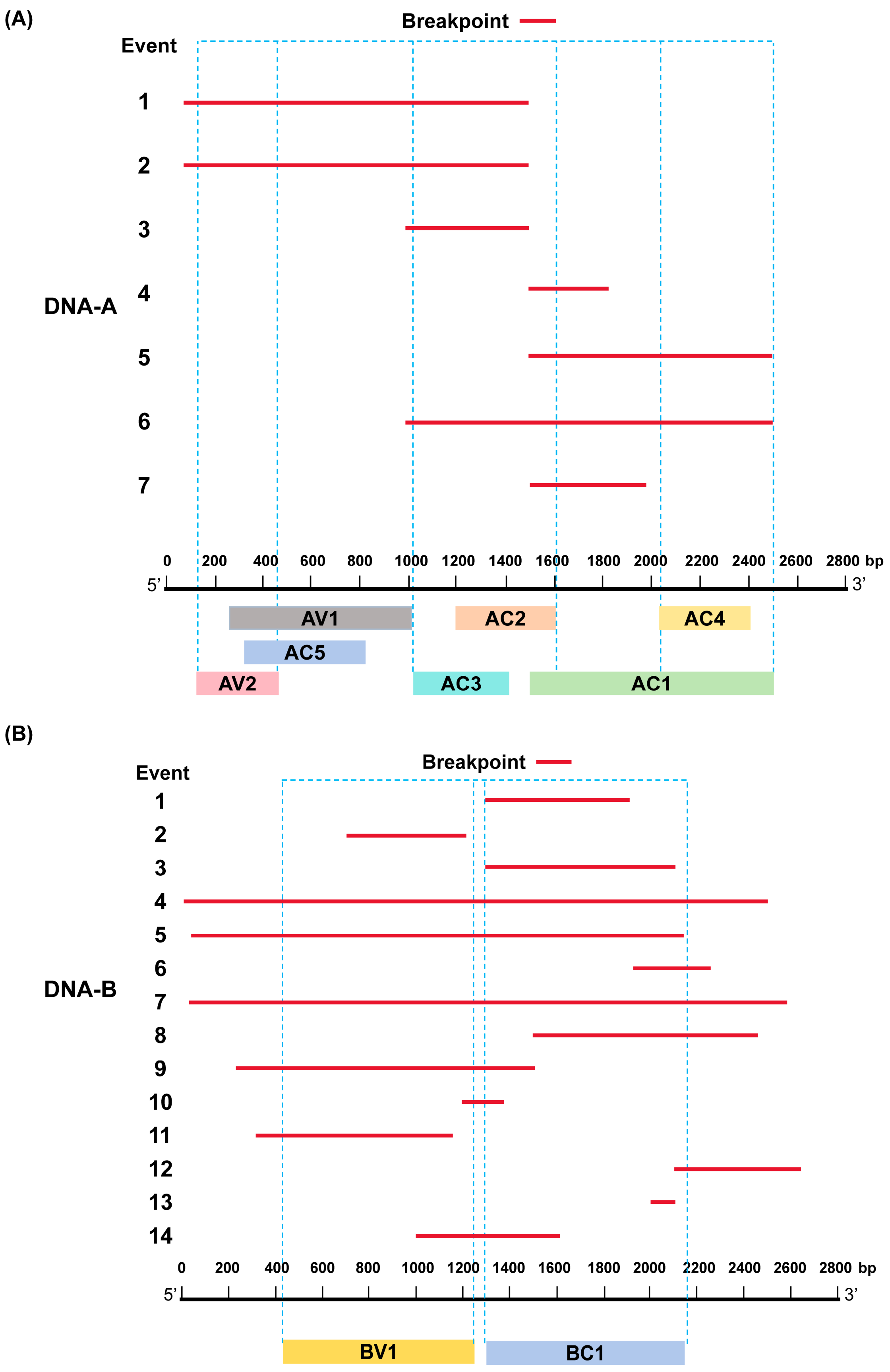

Figure S2. Putative recombination breakpoints in the DNA-A (A) and DNA-B (B) components of SLCCNV strains.

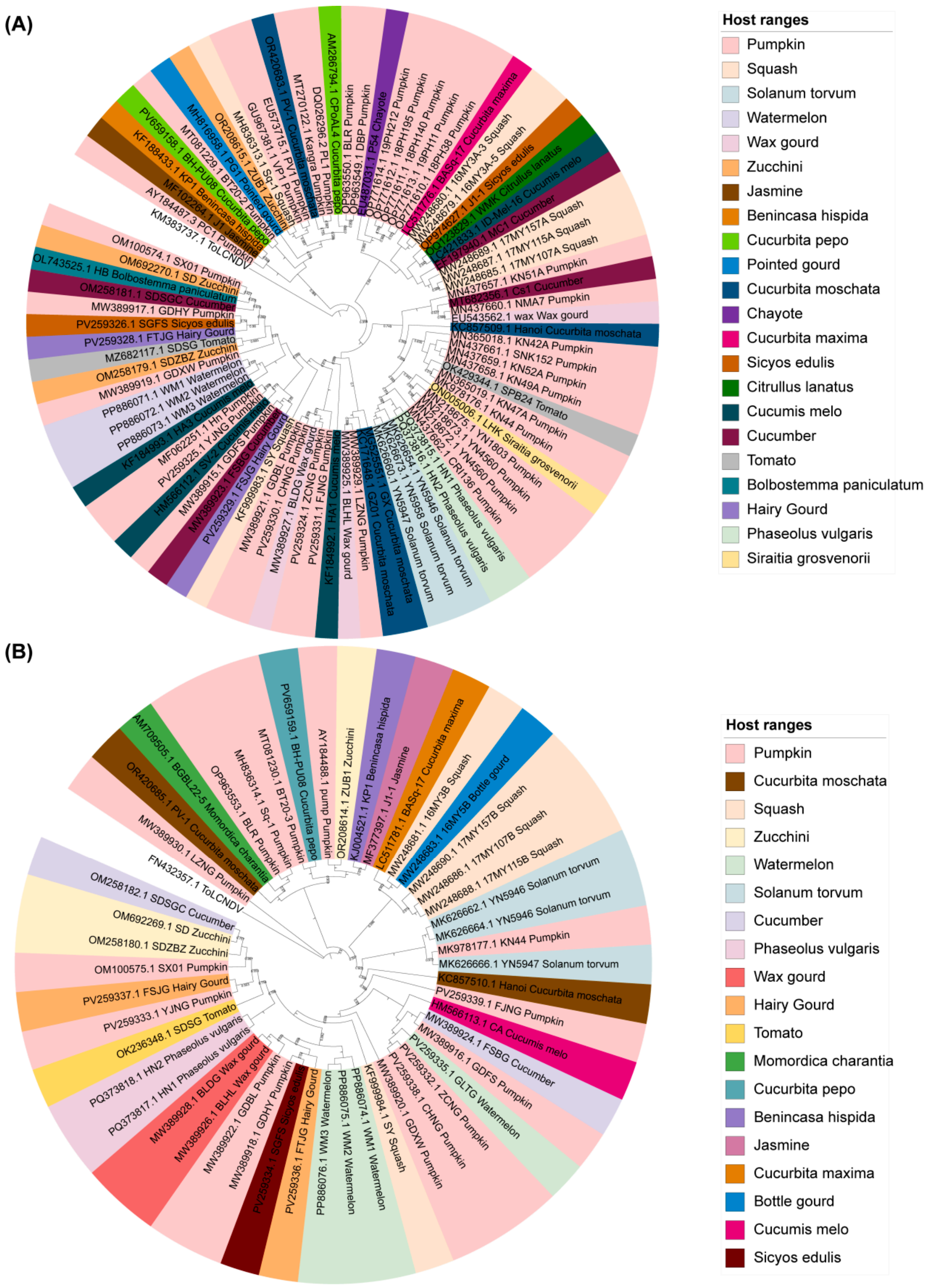

Figure S3. Phylogenetic trees of SLCCNV strains colored by host species. (A) Maximum likelihood phylogenetic tree was constructed using MEGA12 based on 94 SLCCNV DNA-A strains. (B) Maximum likelihood phylogenetic tree was constructed using MEGA12 based on 53 SLCCNV DNA-B strains. Different colors are represented host.

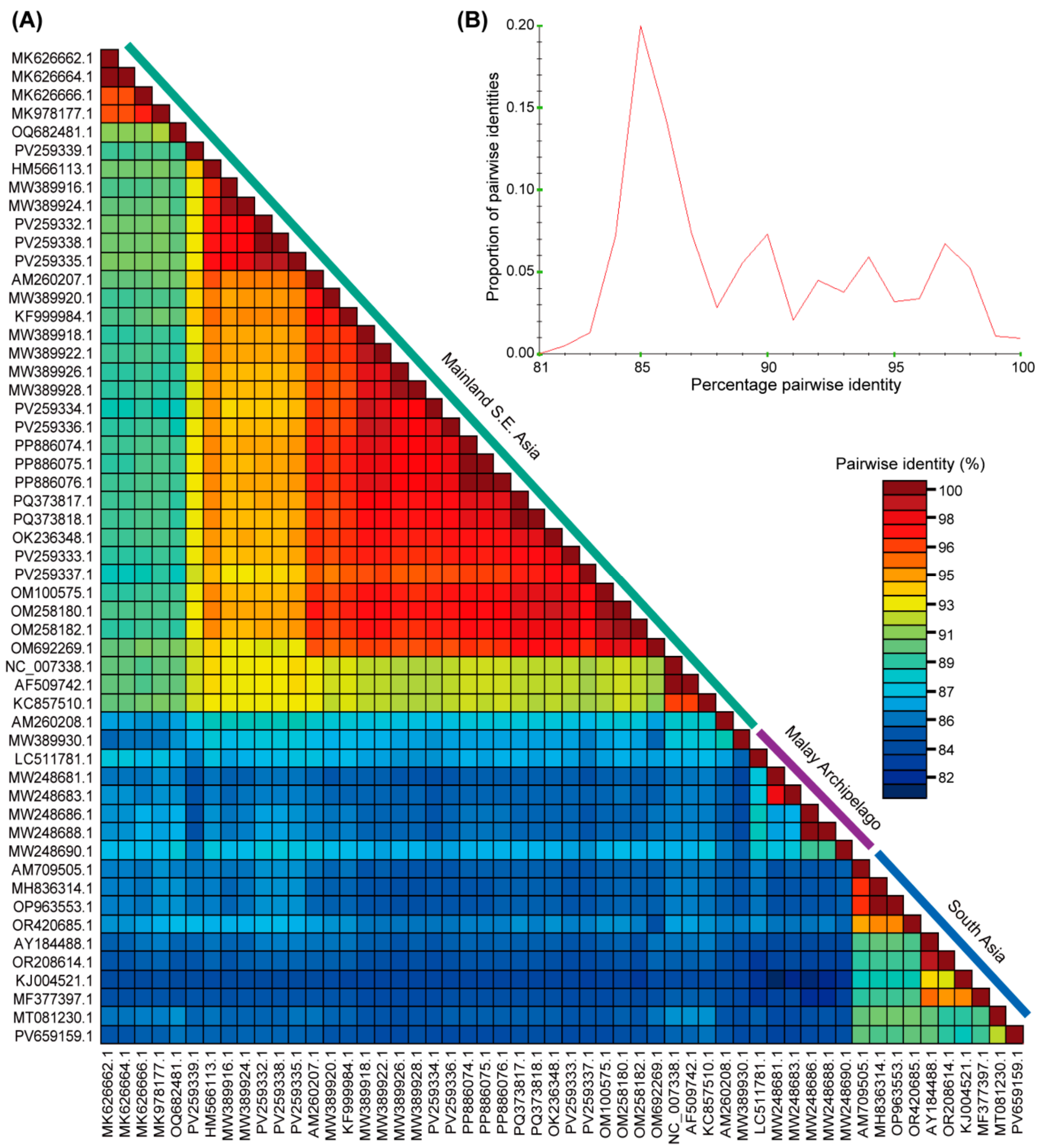

Figure S4. Nucleotide sequence identity analysis of SLCCNV DNA-B strains. (A) Pairwise nucleotide sequence identity among SLCCNV DNA-B strains. The color scale indicates the percentage of nucleotide identity between sequences. (B) Pairwise sequence identity distribution of SLCCNV DNA-B strains.
