# Supplementary material for: Phylogeographic Structure and Molecular Evolution of Squash Leaf Curl China Virus
Source: Viruses. 2026 Jul 19;18(7):794. doi: 10.3390/v18070794 (PMC13431508; doi:10.3390/v18070794)
Supplement: Supplementary file 1 [file viruses-18-00794-s001.zip › Supplementary Tables.pdf]

Table S1. Information for DNA-A strains of SLCCNV used in this study.

No.	Strain [a]	GenBank acc. No.	Region	Host	Sample collection time
1	pGEM-T	AB027465.1			
2	A	AB330078.1	Thailand		
3	K	AF509741.1	Viet Nam		
4	B	AF509743.1	Viet Nam		
5	Hn61	AM260205.1			
6	G25	AM260206.1			
7	CPoAL4	AM286794.1	Pakistan: Lahore	Zucchini (*Cucurbita pepo*)	2005
8	PC1	AY184487.3	India: Coimbatore	Pumpkin (*Cucurbita moschata*)	
9	PL1	DQ026296.2		Pumpkin (*Cucurbita moschata*)	
10	MC1	EF197940.1	Malaysia: Negeri Sembilan	Cucumber (*Cucumis sativus*)	2001
11	P54	EU487031.1	Philippines: Benguet	Chayote (*Sechium edule*)	
12	wax	EU543562.1	Thailand: Nakhon Pathom	Wax gourd (*Benincasa hispida*)	
13	PV1	EU573715.1	India	Pumpkin (*Cucurbita moschata*)	
14	VP1	GU967381.1	India	Pumpkin (*Cucurbita moschata*)	2008
15	SV-2	HM566112.1	China	Melon (*Cucumis melo*)	2010
16	Pum	JN587811.1	India	Pumpkin (*Cucurbita moschata*)	2010
17	GZ01	KC171648.1	China	Winter squash (*Cucurbita moschata*)	2011
18	Hanoi	KC857509.1	Viet Nam	Winter squash (*Cucurbita moschata*)	2012
19	HA1	KF184992.1	China	Melon (*Cucumis melo*)	2012
20	HA3	KF184993.1	China	Melon (*Cucumis melo*)	2012
21	KP1	KF188433.1	India	Wax gourd (*Benincasa hispida*)	2013
22	SY	KF999983.1	China: Hainan	*Squash (Cucurbita pepo)*	2013
23	SV-1	LC417095.1	Indonesia: Yogyakarta	Melon (*Cucumis melo*)	2016
24	ID-Mel-16	LC421833.1	Indonesia: Yogyakarta	Melon (*Cucumis melo*)	2016
25	BASq-17	LC511776.1	Indonesia	Giant pumpkin (*Cucurbita maxima*)	2017
26	Hn	MF062251.1	China	Pumpkin (*Cucurbita moschata*)	
27	J1	MF102264.1	India: Tamil Nadu	Jasmine (*Jasminum sambac*)	2015
28	GX	MG525551.1	China	Winter squash (*Cucurbita moschata*)	2017
29	PG1	MH816958.1	India: Utter Pradesh	Pointed gourd (*Trichosanthes dioica*)	2017
30	Sq-1	MH836313.1	India: Utter Pradesh	Squash (*Cucurbita pepo*)	2017
31	Vir-6569	MK064240.1	Cambodia		2016
32	YN5946	MK626654.1	China: Yunnan	Water eggplant (*Solanum torvum*)	2017
33	YN5947	MK626660.1	China: Yunnan	Water eggplant (*Solanum torvum*)	2017
34	YN5958	MK626673.1	China: Yunnan	Water eggplant (*Solanum torvum*)	2017
35	KN44	MK978176.1	Thailand: Kamphaengsaen district	Pumpkin (*Cucurbita moschata*)	2018
36	YN4560	MN218672.1	China: Yunnan	Pumpkin (*Cucurbita moschata*)	2014
37	YN4560	MN218673.1	China: Yunnan	Pumpkin (*Cucurbita moschata*)	2014
38	YN1803	MN218675.1	China: Yunnan	Pumpkin (*Cucurbita moschata*)	2011
39	KN42A	MN365018.1	Thailand	Pumpkin (*Cucurbita moschata*)	2016
40	KN47A	MN365019.1	Thailand	Pumpkin (*Cucurbita moschata*)	2016
41	KN51A	MN437657.1	Thailand	Pumpkin (*Cucurbita moschata*)	2016

42	KN49A	MN437658.1	Thailand	Pumpkin (*Cucurbita moschata*)	2016
43	KN52A	MN437659.1	Thailand	Pumpkin (*Cucurbita moschata*)	2016
44	NMA7	MN437660.1	Thailand	Pumpkin (*Cucurbita moschata*)	2018
45	SNK152	MN437661.1	Thailand	Pumpkin (*Cucurbita moschata*)	2018
46	CRI136	MN437662.1	Thailand	Pumpkin (*Cucurbita moschata*)	2017
47	J1	MN594504.1	India: Tamil Nadu	Jasmine (*Jasminum sambac*)	
48	BT20-2	MT081229.1	Bangladesh	Pumpkin (*Cucurbita moschata*)	2019
49	Kangra	MT270122.1	India	Pumpkin (*Cucurbita moschata*)	
50	Cs1	MT682356.1	Cambodia: Bakong_Pasat	Cucumber (*Cucumis sativus*)	2012
51	16MY3A-5	MW248679.1	Malaysia	Squash (*Cucurbita pepo*)	2016
52	16MY3A-3	MW248680.1	Malaysia	Squash (*Cucurbita pepo*)	2016
53	16MY5A	MW248682.1	Malaysia	Bottle gourd (*Lagenaria siceraria*)	2016
54	17MY107A	MW248685.1	Malaysia	Squash (*Cucurbita pepo*)	2017
55	17MY115A	MW248687.1	Malaysia	Squash (*Cucurbita pepo*)	2017
56	17MY157A	MW248689.1	Malaysia	Squash (*Cucurbita pepo*)	2017
57	GDFS	MW389915.1	China: Guangdong	Pumpkin (*Cucurbita moschata*)	2019
58	GDHY	MW389917.1	China: Guangdong	Pumpkin (*Cucurbita moschata*)	2020
59	GDXW	MW389919.1	China: Guangdong	Pumpkin (*Cucurbita moschata*)	2019
60	GDBL	MW389921.1	China: Guangdong	Pumpkin (*Cucurbita moschata*)	2019
61	FSBG	MW389923.1	China: Guangdong	Cucumber (*Cucumis sativus*)	2019
62	BLHL	MW389925.1	China: Guangdong	Wax gourd (*Benincasa hispida*)	2019
63	BLDG	MW389927.1	China: Guangdong	Wax gourd (*Benincasa hispida*)	2019
64	LZNG	MW389929.1	China: Guangdong	Pumpkin (*Cucurbita moschata*)	2019
65	SDSG	MZ682117.1	China: Shandong	Tomato (*Solanum lycopersicum*)	2020
66	B1	NC_007339.1	Viet Nam		
67	SPB24	OK429344.1	Thailand	Tomato (*Solanum lycopersicum*)	2020
68	HB	OL743525.1	China: Hebei	Jiabeimu（*Bolbostemma paniculatum*）	2020
69	WF-10	OM049535.1	India: Utter Pradesh		2016
70	WF-26	OM049536.1	India: Utter Pradesh		2016
71	WF-32	OM049537.1	India: Utter Pradesh		2016
72	SX01	OM100574.1	China	Pumpkin (*Cucurbita moschata*)	2020
73	SDZBZ	OM258179.1	China: Shandong	Zucchini (*Cucurbita pepo*)	2020
74	SDSGC	OM258181.1	China: Guangdong	Cucumber (*Cucumis sativus*)	2020
75	SD	OM692270.1	China	Zucchini (*Cucurbita pepo*)	2020
76	LHK	ON005006.1	China: Guangxi	Monk fruit (*Siraitia grosvenorii*)	2021
77	18PH38	OP771610.1	Philippines	Pumpkin (*Cucurbita moschata*)	2018
78	18PH140	OP771611.1	Philippines	Pumpkin (*Cucurbita moschata*)	2018
79	18PH195	OP771612.1	Philippines	Pumpkin (*Cucurbita moschata*)	2018
80	19PH11	OP771613.1	Philippines	Pumpkin (*Cucurbita moschata*)	2019
81	19PH212	OP771614.1	Philippines	Pumpkin (*Cucurbita moschata*)	2019
82	TMK	OP963548.1	India: Karnataka	Pumpkin (*Cucurbita moschata*)	2021
83	DBP	OP963549.1	India: Karnataka	Pumpkin (*Cucurbita moschata*)	2021
84	BLR	OP963550.1	India: Karnataka	Pumpkin (*Cucurbita moschata*)	2021
85	J1-1	OP974627.1	Indonesia	Chayote (*Sechium edule*)	2019

86	WMK	OQ123829.1	Indonesia		2021
87	ZUB1	OR208615.1	India: Karnataka	Zucchini (*Cucurbita pepo*)	2021
88	PV-1	OR420683.1	India: New Delhi	Winter squash (*Cucurbita moschata*)	2021
89	WM1	PP886071.1	China: Zhejiang	Watermelon (*Citrullus lanatus*)	2023
90	WM2	PP886072.1	China: Zhejiang	Watermelon (*Citrullus lanatus*)	2023
91	WM3	PP886073.1	China: Zhejiang	Watermelon (*Citrullus lanatus*)	2023
92	HN1	PQ373815.1	China: Henan	Common Bean (*Phaseolus vulgaris*)	2024
93	HN2	PQ373816.1	China: Henan	Common Bean (*Phaseolus vulgaris*)	2024
94	ZCNG	PV259324.1	China: Guangdong	Pumpkin (*Cucurbita moschata*)	2023
95	YJNG	PV259325.1	China: Guangdong	Pumpkin (*Cucurbita moschata*)	2024
96	SGFS	PV259326.1	China: Guangdong	Chayote (*Sechium edule*)	2024
97	FTJG	PV259328.1	China: Guangdong	Hairy Gourd (*Benincasa hispida*)	2024
98	FSJG	PV259329.1	China: Guangdong	Hairy Gourd (*Benincasa hispida*)	2024
99	CHNG	PV259330.1	China: Guangdong	Pumpkin (*Cucurbita moschata*)	2023
100	FJNG	PV259331.1	China: Guangdong	Pumpkin (*Cucurbita moschata*)	2024
101	BH-PU08	PV659158.1	Bhutan: Samtenling	Zucchini (*Cucurbita pepo*)	2023

Table S2. Information for DNA-B strains of SLCCNV used in this study.

No.	Strain [a]	GenBank acc. No.	Region	Host	Sample collection time
1	Hn61	AM260207.1			
2	G25	AM260208.1			
3	BGBL22-5	AM709505.1	Pakistan: Lahore	Bitter Gourd (*Momordica charantia*)	2004
4	CPoBL2	AM778959.1	Pakistan: Lahore	Zucchini (*Cucurbita pepo*)	2005
5	pump	AY184488.1	India: Coimbatore	Pumpkin (*Cucurbita moschata*)	
6	Varanasi-1	FJ859881.1	India	Pumpkin (*Cucurbita moschata*)	
7	Varanasi	GU967382.1	India	Pumpkin (*Cucurbita moschata*)	2008
8	CA	HM566113.1	China	Melon (*Cucumis melo*)	2010
9	Pum	JN624306.1	India	Pumpkin (*Cucurbita moschata*)	2010
10	GZ01	KC171649.1	China	Pumpkin (*Cucurbita moschata*)	2011
11	Hanoi	KC857510.1	Viet Nam	Pumpkin (*Cucurbita moschata*)	2012
12	SY	KF999984.1	China: Hainan	*Squash* (*Cucurbita pepo*)	2013
13	KP1	KJ004521.1	India: Perambalur	Hairy Gourd (*Benincasa hispida*)	2013
14	BASq-17	LC511781.1	Indonesia	Giant pumpkin (*Cucurbita maxima*)	2017
15	Hn	MF062252.1	China	Pumpkin (*Cucurbita moschata*)	
16	J1-1	MF377397.1	India: Tamilnadu	Jasmine (*Jasminum sambac*)	2015
17	GX2017	MG525552.1	China	Pumpkin (*Cucurbita moschata*)	2017
18	PG1	MH816957.1	India: Utter Pradesh	Pointed gourd (*Trichosanthes dioica*)	2017
19	Sq-1	MH836314.1	India: Utter Pradesh	Pumpkin (*Cucurbita moschata*)	2017
20	YN5946	MK626662.1	China: Yunnan	Turkey berry (*Solanum torvum*)	2017
21	YN5946	MK626664.1	China: Yunnan	Turkey berry (*Solanum torvum*)	2017
22	YN5947	MK626666.1	China: Yunnan	Turkey berry (*Solanum torvum*)	2017
23	KN44	MK978177.1	Thailand: Kamphaengsaen district	Pumpkin (*Cucurbita moschata*)	2018
24	J1	MN594505.1	India: Tamil Nadu	Jasmine (*Jasminum sambac*)	
25	BT20-3	MT081230.1	Bangladesh	Pumpkin (*Cucurbita moschata*)	2019
26	Cs1	MT682357.1	Cambodia: Bakong_Pasat	Cucumber (*Cucumis sativus*)	2012
27	16MY3B	MW248681.1	Malaysia	Squash (*Cucurbita pepo*)	2016
28	16MY5B	MW248683.1	Malaysia	Bottle gourd (*Lagenaria siceraria*)	2016
29	17MY85B	MW248684.1	Malaysia		2017
30	17MY107B	MW248686.1	Malaysia	Squash (*Cucurbita pepo*)	2017
31	17MY115B	MW248688.1	Malaysia	Squash (*Cucurbita pepo*)	2017
32	17MY157B	MW248690.1	Malaysia	Squash (*Cucurbita pepo*)	2017
33	GDFS	MW389916.1	China: Guangdong	Pumpkin (*Cucurbita moschata*)	2019
34	GDHY	MW389918.1	China: Guangdong	Pumpkin (*Cucurbita moschata*)	2020
35	GDXW	MW389920.1	China: Guangdong	Pumpkin (*Cucurbita moschata*)	2019
36	GDBL	MW389922.1	China: Guangdong	Pumpkin (*Cucurbita moschata*)	2019
37	FSBG	MW389924.1	China: Guangdong	Cucumber (*Cucumis sativus*)	2019
38	BLHL	MW389926.1	China: Guangdong	Wax gourd (*Benincasa hispida*)	2019
39	BLDG	MW389928.1	China: Guangdong	Wax gourd (*Benincasa hispida*)	2019
40	LZNG	MW389930.1	China: Guangdong	Pumpkin (*Cucurbita moschata*)	2019

41	B1	NC_007338.1	Viet Nam		
42	SDSG	OK236348.1	China: Shandong	Tomato (*Solanum lycopersicum*)	2020
43	SX01	OM100575.1	China	Pumpkin (*Cucurbita moschata*)	2020
44	SDZBZ	OM258180.1	China: Shandong	Zucchini (*Cucurbita pepo*)	2020
45	SDSGC	OM258182.1	China: Guangdong	Cucumber (*Cucumis sativus*)	2020
46	SD	OM692269.1	China: China	Zucchini (*Cucurbita pepo*)	2020
47	DTMK	OP963551.1	India: Karnataka	Pumpkin (*Cucurbita moschata*)	2021
48	DBP	OP963552.1	India: Karnataka	Pumpkin (*Cucurbita moschata*)	2021
49	BLR	OP963553.1	India: Karnataka	Pumpkin (*Cucurbita moschata*)	2021
50	LHG	OQ682481.1	China: Guangxi		2022
51	ZUB1	OR208614.1	India	Zucchini (*Cucurbita pepo*)	2021
52	PV-1	OR420685.1	India: New Delhi	Pumpkin (*Cucurbita moschata*)	2021
53	KM2	OR860426.1	India: Coimbatore	Pumpkin (*Cucurbita moschata*)	2023
54	WM1	PP886074.1	China: Zhejiang	Watermelon (*Citrullus lanatus*)	2023
55	WM2	PP886075.1	China: Zhejiang	Watermelon (*Citrullus lanatus*)	2023
56	WM3	PP886076.1	China: Zhejiang	Watermelon (*Citrullus lanatus*)	2023
57	HN1	PQ373817.1	China: Henan	Common Bean (*Phaseolus vulgaris*)	2024
58	HN2	PQ373818.1	China: Henan	Common Bean (*Phaseolus vulgaris*)	2024
59	ZCNG	PV259332.1	China: Guangdong	Pumpkin (*Cucurbita moschata*)	2023
60	YJNG	PV259333.1	China: Guangdong	Pumpkin (*Cucurbita moschata*)	2024
61	SGFS	PV259334.1	China: Guangdong	Chayote (*Sechium edule*)	2024
62	GLTG	PV259335.1	China: Guangdong	Watermelon (*Citrullus lanatus*)	2024
63	FTJG	PV259336.1	China: Guangdong	Hairy Gourd (*Benincasa hispida*)	2024
64	FSJG	PV259337.1	China: Guangdong	Hairy Gourd (*Benincasa hispida*)	2024
65	CHNG	PV259338.1	China: Guangdong	Pumpkin (*Cucurbita moschata*)	2023
66	FJNG	PV259339.1	China: Guangdong	Pumpkin (*Cucurbita moschata*)	2024
67	BH-PU08	PV659159.1	Bhutan: Samtenling	Zucchini (*Cucurbita pepo*)	2023

Table S3. InDels events in individual proteins of SLCCNV strains.

Event	Protein [a]	Position (amid acid)	GenBank acc.no.
I	AC1 (Rep)	Δ211-216	AM286794.1, DQ026296.2, MT270122.1, OP963549.1, OP963550.1
II	AC1 (Rep)	Δ253	EU543562.1, MN437657.1, MN437660.1
III	AC3 (Ren)	Δ83	EU487031.1
IV	AC5 (VSR)	Δ169-195	MK626660.1, MK626673.1
V	AV1 (CP)	Δ15	EU543562.1, MN437657.1, MN437660.1
VI	AV2 (Pre-CP)	Δ94	EU543562.1, MN437660.1, MN437657.1
VII	BC1(MP)	Δ155-166	PV259332.1, PV259338.1
VIII	BC1(MP)	Δ278-289	MW389918.1

[a] AC1 = Replication-associated protein, AC3 = Replication enhancer protein, AC5 = RNA silencing suppressor and virulence determinant protein, AV1 = Coat protein, AV2 = Pre-coat protein, BC1 = Movement protein.
